# Preparation of Transglutaminase-Catalyzed Rice Bran Protein Emulsion Gels as a Curcumin Vehicle

**DOI:** 10.3390/foods13132072

**Published:** 2024-06-29

**Authors:** Jie Liu, Siqi Yang, Jiayuan Liu, Hongzhi Liu, Ziyuan Wang

**Affiliations:** 1 National Center of Technology Innovation for Grain Industry (Comprehensive Utilization of Edible By-Products), Beijing Technology and Business University, Beijing 100048, China; 2 Key Laboratory of Geriatric Nutrition and Health (Beijing Technology and Business University), Ministry of Education, Beijing 100048, China

**Keywords:** rice bran protein, emulsion gel, transglutaminase, entrapment efficiency

## Abstract

Protein-based emulsion gels have tunable viscoelasticity that can be applied to improve the stability of bioactive ingredients. As the by-product of rice processing, rice bran protein (RBP) has high nutritional value and good digestibility, exhibiting unique value in the development of hypoallergenic formula. In this study, the effect of transglutaminase (TGase) cross-linking on the physicochemical properties of RBP emulsion gels was investigated. To improve the stability of curcumin against environmental stress, the entrapment efficiency and stability of curcumin in the emulsion gel systems were also evaluated. The results indicated that TGase increased the viscoelastic modulus of RBP emulsion gels, resulting in a solid-like structure. Moreover, the entrapment efficiency of curcumin was increased to 93.73% after adding TGase. The thermal stability and photo-stability of curcumin were enhanced to 79.54% and 85.87%, respectively, compared with the sample without TGase addition. The FTIR results showed that TGase induced the cross-linking of protein molecules and the secondary structure change in RBP. Additionally, SEM observation confirmed that the incorporation of TGase promoted the formation of a compact network structure. This study demonstrated the potential of RBP emulsion gels in protecting curcumin and might provide an alternative strategy to stabilize functional ingredients.

## 1. Introduction

The rice processing industry produces a large number of by-products, among which rice bran constitutes around 10% [1]. Moreover, rice bran rich in protein (10–16%) exhibits excellent nutritional value and health benefits, giving it great potential to be used in functional food development [2]. In particular, rice bran protein (RBP) with good digestibility, exhibits unique value in hypoallergenic formula development [1]. RBP has a high content of lysine and threonine compared to other cereal proteins and an excellent amino acid composition, making it suitable for infant food. For emulsion processing, RBP has good emulsification properties that could be used as a natural macromolecular emulsion stabilizer [3]. However, RBP is currently underutilized, which results in a waste of food resources [4]. Therefore, it is necessary to investigate feasible processing techniques to improve the properties of RBP and expand its application in the food industry.

Emulsion gels are soft solid-like food systems that exist as both emulsions and gels [5]. Protein-stabilized emulsions with low or medium oil content can form emulsion gels by common food processing treatments such as heating, acidification, and enzymatic action [6]. Commonly used proteins are soy protein, whey protein, corn alkyd protein, and myofibrillar protein, while RBP is underutilized in emulsion gel production [7,8,9]. Protein-based emulsion gels have tunable viscoelasticity and can be used to deliver and improve the stability of bioactive substances [10]. The appearance, texture, and stability of emulsion gels mainly depend on their structural type, material concentration, and intermolecular interactions. For instance, the concentration of oil droplets, protein particles, and crosslinkers in emulsion could affect the aggregation degree of these particles and the characteristics of emulsion gels [11]. Therefore, the functional properties of emulsion gels could be tailored by selecting appropriate material ratios and processing conditions.

Transglutaminase (TGase) cross-linking is an efficient way of preparing emulsion gels as it can catalyze the acyl transfer reaction between lysine and glutamyl residues, forming solid emulsion gels by covalent cross-linking [12]. It has been found that TGase cross-linking provides assistance in improving the freeze–thaw stability and enhancing the gelling properties of mung bean protein emulsions [13]. The addition of TGase also played a positive role in the entrapment of bioactive ingredients. For instance, TGase-induced whey protein isolation–milk fat emulsion gel exhibited good physical stability and high entrapment efficiency for lutein [14].

Curcumin is a natural fat-soluble polyphenol with a variety of physiologically active functions. However, the heat and light sensitivity of curcumin has limited its application in the food industry [15,16]. Thus, the construction of process-moderated delivery systems is needed to enhance its storage stability and bioavailability. Su et al. [17] prepared emulsion gels with intestinal slow-release function by using whey protein isolate and κ-carrageenan, which significantly delayed the release of curcumin in the gastric environment and facilitated the intestinal absorption of curcumin. However, the application of RBP emulsion gels for the entrapment and stabilization of curcumin by using TGase cross-linking has not been reported yet.

In this study, we aimed to investigate the role of TGase cross-linking on the textural, rheological, and functional properties of surfactant-free RBP emulsions and emulsion gels. The entrapment efficiency and stabilization effect on curcumin by using this emulsion gel have also been investigated.

## 2. Materials and Methods

### 2.1. Materials

Rice bran protein (protein 90% *w*/*w*) was obtained from Xi’an Nansi Biotechnology Co., Ltd. (Xi’an, China). Transglutaminase (activity, 120 U/g) was obtained from Jiangsu Yiming Biological Technology Co., Ltd. (Jiangsu, China). Soybean oil was obtained from Yihai Kerry Arawana Holdings Co., Ltd. (Shanghai, China). Curcumin was purchased from Shanghai Yuanye Bio-Technology Co., Ltd. (Shanghai, China). Unless otherwise stated, all reagents used in this study were of analytical grade.

### 2.2. Preparation of RBP Emulsions

RBP solution (5%, *w*/*v*) was stirred at room temperature for 2 h and then placed at 4 °C for 12 h to be fully hydrated. Then, soybean oil (Φ = 0.2, *v/v*) was added to RBP solution. The RBP emulsion was obtained after stirring at 10,000 rpm for 2 min using a high-shear mixer (PT2500E, Kinematica, Luzern, Switzerland) followed by sonicating in an ice bath using a multi-purpose constant temperature ultrasonic extractor (BILON-1000CT, Shanghai Bilon Instrument Co., Ltd., Shanghai, China). The sonication process lasted 15 min by using a 3 mm diameter probe, with 3 s of sonication and 2 s of rest (the volume of emulsion: 20 mL, power: 300 W, frequency: 20 kHz). After sonication, different doses of TGase (0, 10, 20, 30, and 40 U/g RBP) were added. Then, the emulsions were stored in a refrigerator at 4 °C.

### 2.3. Characterization of RBP Emulsions

The ζ-potential, average particle size, and PDI of the emulsions were measured using a Nano laser particle size analyzer (ZS-90, Malvern Instruments, Malvern, UK). To minimize the effects of multiple scattering, the RBP emulsion prepared in Section 2.2 was diluted 100 times with deionized water and mixed well before measurement [18]. All measurements were carried out at 25 °C, and the results were reported as averages of three readings.

### 2.4. Preparation of RBP Emulsion Gels

For the preparation of emulsion gels, different doses of TGase (0, 10, 20, 30, and 40 U/g RBP) were added to RBP emulsions, respectively, and incubated at 37 °C for 12 h [19]. After cooling at room temperature, the emulsion gels were stored at 4 °C.

### 2.5. Characterization of RBP Emulsion Gels

#### 2.5.1. Dynamic Rheological Analysis

The rheological properties of RBP emulsion gels were analyzed using a rheometer (HR-1, Discovery, New Castle, DE, USA). The tests were carried out at 25 °C. A parallel plate of 40 mm diameter was used with a measurement gap of 1 mm and parameter settings based on previous studies [20]. Changes in storage modulus (G′), loss modulus (G″), and viscosity were measured. The storage modulus (G′) and loss modulus (G″) of emulsion gels were detected at the constant strain of 0.5% and the scanning frequency from 0.1 to 100 rad/s. Strain scanning and a shear rate scan were used to test the linear viscoelastic region and the apparent viscosity of the emulsion gels at a frequency of 1 Hz.

#### 2.5.2. Textural Properties Analysis

The RBP emulsion gels (20 mL) were prepared in 25 mL beakers for texture profile analysis (TPA). A texture analyzer (CT3, Brookfield, Middleboro, MA, USA) was used to investigate the texture properties of the RBP emulsion gels, and was equipped with a cylindrical probe (TA44). Test speed and compression ratio were 1 mm/s and 50%, respectively. Residence time of 5 s at room temperature (25 °C).

#### 2.5.3. Fourier-Transform Infrared (FTIR) Analysis

FTIR observation was conducted based on the method described by Liu et al. [21]. The secondary structure of RBP emulsion gels was determined by using an infrared spectroscope (Thermo scientific NICOLET IS10, Thermo Fisher Scientific, Waltham, MA, USA). The spectrum in the range of 4000 to 400 cm^−1^ was collected with 32 scans. The secondary structural changes in protein gel samples were calculated using OMNIC 9.2 software.

#### 2.5.4. X-ray Diffraction (XRD)

The XRD patterns of the samples were examined according to a previously reported method [22]. The powder of freeze-dried RBP emulsion gels was characterized by a D8 Advance X-ray diffractometer (D8 ADVANCE, Bruker, Karlsruhe, Germany). Diffraction angles 2θ were measured over the range 5–50° by using Cu Kα radiation with λ = 0.154 nm.

#### 2.5.5. Scanning Electron Microscope (SEM)

The microstructure of RBP emulsion gel was observed according to the method described by Wang et al. [23]. Before observation, the defatted samples were fully dried and sprayed with gold. Photographs were taken at 1000× magnification.

### 2.6. Construction of Curcumin-Loaded Emulsions and Emulsion Gels

For the preparation of curcumin-loaded emulsions and emulsion gels, curcumin (0.3%, *w*/*v*) was dissolved into the soybean oil. Then, curcumin solution (oil phase) was mixed with RBP solution as described in Section 2.2 and Section 2.4. The whole process was carried out in a light-proof environment and the emulsion gels were stored at 4 °C.

### 2.7. Entrapment Efficiency of Curcumin in RBP Emulsion Gel

The curcumin concentrations of the emulsion and emulsion gels were determined according to a reported method with slight modifications [24]. Different concentrations of curcumin ethanol solution were prepared, and their absorbance at 425 nm was determined to make a standard curve. Curcumin entrapped by the sample was recovered by adding ethanol (9 mL) to the emulsion (1 mL) and emulsion gel (1 g). The mixture was centrifuged at 6000 rpm for 10 min, and the supernatant was collected and tested by a microplate reader (Synergy H1, Bio Tek, Winooski, VT, USA). The entrapment efficiency of curcumin was calculated using Equation (1):

EE (%) = C_0_/C_1_ × 100% (1)

where EE represents the entrapment efficiency of curcumin; C_0_ is the concentration of curcumin entrapped by the emulsion gels (%); C_1_ is the total curcumin concentration in the sample (%).

### 2.8. Stability of Curcumin in RBP Emulsion Gel

#### 2.8.1. Thermal Stability of Curcumin

The curcumin-loaded emulsion gel was heated at different temperatures (70 °C, 80 °C, 90 °C, and 100 °C, respectively) for 30 min and then immediately cooled down. Then, the emulsion gel was mechanically mashed [25]. The same procedure was used to test curcumin solution as the control. The curcumin concentration after heat treatment was determined as described in Section 2.5. The thermal stability (TS) of curcumin was determined by using the Equation (2):

TS = C_2_/C_3_ × 100% (2)

where C_2_ and C_3_ were the curcumin concentrations with and without heat treatment, respectively (%).

#### 2.8.2. Photo-Stability of Curcumin

The photo-stability of curcumin in RBP emulsion gels was examined according to a reported method [20]. The emulsion gel (1 g) was placed in a 15 mL centrifuge tube and exposed to UV light (35 W, 385 nm) for 3 h, 6 h, 9 h, and 12 h, respectively. The curcumin content was determined as described in Section 2.5. The photo-stability (PS) of curcumin was calculated by Equation (3):

PS = C_4_/C_5_ × 100% (3)

where C_4_ and C_5_ were the curcumin concentrations after and before UV illumination, respectively (%).

### 2.9. Statistical Analysis

Experimental data were statistically analyzed by the SPSS 27.0.1 (SPSS Inc., Chicago, IL, USA). One-way analysis of variance (ANOVA) with Duncan’s post hoc test was used to determine significant differences, and differences were considered significant at *p* < 0.05.

## 3. Results and Discussion

### 3.1. Characterization of RBP Emulsions

**Figure 1:**
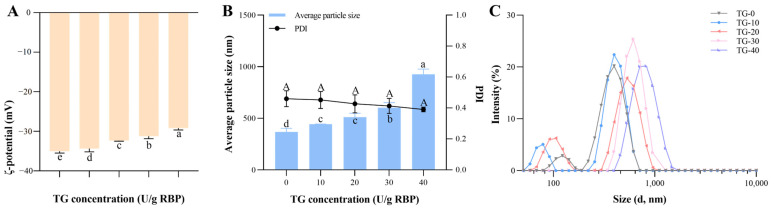
Influence of different concentrations of TGase on (**A**) ζ-potential. Different letters are significantly different (*p* < 0.05). (**B**) average particle size and PDI. Different uppercase letters denote significant difference of PDI and different lowercase letters denote significant difference of average particle size (*p* < 0.05). (**C**) particle size distribution of RBP emulsions.

ζ-potential is a useful parameter in gel formation because electrostatic charge is a key factor in polymer interactions [26]. As shown in Figure 1A, the absolute value of ζ-potential significantly decreased with the addition of TGase (*p* < 0.05). In Figure 1B,C, as the enzyme amount increased, the particle size also showed a significant increase compared to the control, and the protein particle size distribution was shifted to the right. This might be due to the cross-linking effect of TGase on RBP, thereby reducing the number of smaller protein particles in the system. Cross-linked aggregation of proteins might result in charged amino acids being buried [27]. The decrease in electrostatic repulsion could increase the interaction between molecules at the interface [28]. There was no significant difference in the PDI values of the samples, which indicated that the particle size dispersion of the protein dispersion system was comparable to that of the control. Similar results were observed when investigating the effect of TGase cross-linking on the foaming properties of RBP [27]. The emulsion after enzyme addition showed a decrease in the absolute value of ζ-potential and a significant rightward shift in the particle size distribution.

### 3.2. Rheological Properties of RBP Emulsion Gels

**Figure 2:**
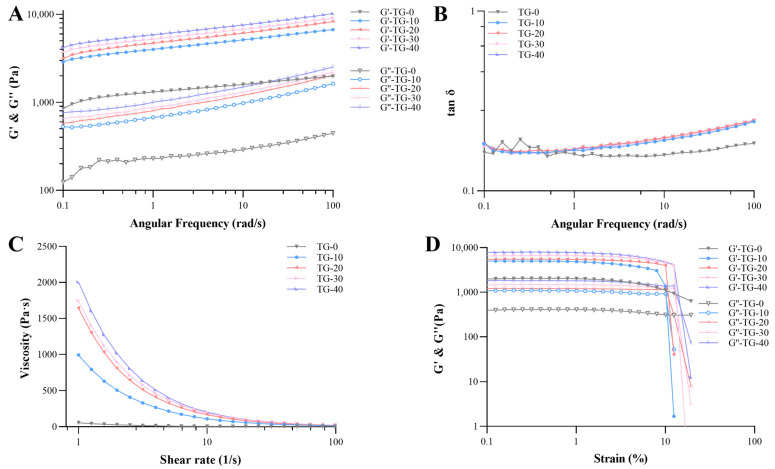
Influence of different concentrations of TGase on the rheological property of RBP emulsion gels. Frequency sweep: (**A**) G′ and G″, (**B**) tan δ, and (**C**) viscosity. Strain scan: (**D**) G′ and G″.

TGase cross-linked emulsion gels showed an increase in viscoelastic modulus (G′ and G″) with the increase in TGase addition, indicating the formation of a stronger gel-like structure (Figure 2A). In general, highly structured solids or gels have elastic rheological behavior (G′ > G″, tan δ < 1) [29]. This is mainly due to the covalent bonds and physical interactions between the proteins adsorbed on the surface of the emulsion droplets and the proteins aggregated in the gel matrix structure [5]. These interactions contributed to the formation of a network structure within the emulsion gel. In Figure 2A, the viscoelastic modulus of RBP emulsion gels had a frequency-dependent behavior. Xie et al. explored the effect of γ-polyglutamic acid (γ-PGA) addition on the rheology properties of fish gelatin (FG) emulsion gels and observed similar results [30]. This indicated that chain entanglements and macromolecule connections in emulsion gels could be disrupted by applying high shear rates. The tan δ values were less than one for different samples, indicating that the generated emulsion gels were elastic behavior-dominant gels (Figure 2B). If the tan δ value was higher than 0.1, it indicated that the structure was between highly concentrated biopolymer and real gel [31]. The tan δ values of RBP emulsion gels were lower than 1, but higher than 0.1, which indicated the presence of an elastic structure in a weak biopolymer gel. The emulsion gels were considered soft solids that contained a liquid phase, and the liquid phase was trapped within the pores of a gel network [11,32]. Similar results of emulsion gels have been reported in previous studies [33,34].

In Figure 2C, all emulsion gels displayed shear-thinning behavior with the increase in shear rate. The difference in the viscosity values of the emulsion gels at high shear rates was slight. This result might be because most of the RBP dispersed on the surface of the oil droplets and the cross-linking that occurred between the RBP resulted in the formation of a gel network. Then, with the rapid increase in shear rate, the network structure of the gel was disrupted and collapsed. The droplets in the system were deformed and ruptured, which led to a reduction in flow resistance and viscosity. TGase cross-linking-induced emulsion gels exhibited a higher viscosity than blank samples. One of the reasons might be that the gel network of TGase-induced emulsion gels was more compact and homogeneous. It reduced the accumulation of oil droplets through hydrophobic interaction and hydrogen bonding, which ultimately resulted in lower apparent viscosity [29]. As shown in Figure 2D, G′ was much higher than G″ at low strain amplitudes, and for small deformations, G′ and G″ were almost independent of strain. Moreover, the values of G′ and G″ increased with increasing TGase concentration. Thus, at higher TGase concentrations, all emulsion gels displayed elastically dominant behavior and the stiffness of the emulsion gels increased. With further increase in strain amplitude, both G′ and G″ decreased, with G′ decreasing faster than G″, leading to the observed crossover point. The crossover point was considered an indicator of structural disruption of the particle network, suggesting that the emulsion began to exhibit viscosity-dominated behavior. In addition, the G′ and G″ values of the sonic-assisted TGase-induced emulsion gel were higher than those formed solely through sonication. This result might be because the higher droplet aggregation in the TGase-induced emulsion gels avoided the disruption of the gel network structure. The stable system in TGase-induced BRP gel might also be ascribed to inter- or intramolecular cross-linking due to the acyl transfer reaction between the γ-formamide group of glutamine residues and the ε-amino group of lysine in the proteins [11].

### 3.3. Textural Properties of RBP Emulsion Gels

Different preparation methods might lead to different mechanical properties of protein-based emulsion gels. The textural properties of RBP emulsion gels formed by different concentrations of TGase are shown in Table 1. Hardness refers to the force required for food materials to reach a certain degree of deformation. Chewiness refers to the energy needed to masticate solid food to a state ready for swallowing [35]. Hardness and chewiness reflect the mechanical properties of the gel and provide references for the evaluation of the subjective senses while chewing the gel [36]. TGase enhanced TPA parameters such as the hardness, chewiness, and springiness of RBP emulsion gels. This might be because, under ultrasonic cavitation, the structure of the protein was unfolded and exposed more action sites to TGase. The emulsion gel system was mainly maintained by weak bonds between proteins and TGase promoted the cross-linking of RBP particles on the surface of the droplets, which improved the compaction and stability of the gel and contributed to the increased hardness [25]. Moreover, chewiness and springiness might be affected by the network structure of emulsion gel [37]. TGase can catalyze ε-(γ-glutamy)-lysine crosslinker, enhancing physical interactions and facilitating covalent bonding. The formation of this binding leads to enhanced resistance to external forces along with increased chewiness and springiness [38]. In line with our findings, Tian et al. [39] prepared ovalbumin emulsion gels and reported that TGase-induced protein gels exhibited greater hardness and chewiness by forming a more homogeneous and stable system. In conclusion, due to the cross-linking between the protein particle and the formation of a solid-like structure, the TPA parameters of the RBP emulsion gel were improved.

### 3.4. FTIR

**Figure 3:**
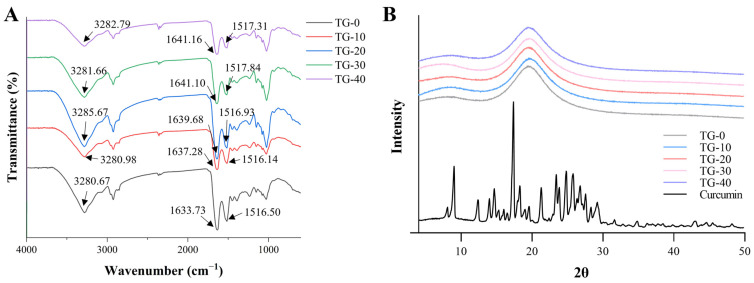
FTIR spectra of RBP emulsion gels (**A**) and XRD of RBP emulsion gels (**B**).

Changes in functional groups and chemical bonding of RBP emulsion gels were detected by FTIR. As shown in Figure 3A, the addition of TGase can alter the secondary structure of RBP in the gel. Generally, the amide I band (1600–1700 cm^−1^) is caused by stretching vibrations of the C-O and C-N groups, and the amide II band (1500–1590 cm^−1^) is caused by bending vibrations of the N-H group and stretching vibrations of the C-N group. The band observed at 3000–3500 cm^−1^ is associated with stretching vibrations of the -OH group [40]. Here, all samples showed similar characteristic peaks, which indicated that TGase treatment did not change the primary structure of RBP. However, the peaks moderately shifted around 3280 cm^−1^ after cross-linking with TGase. This might be due to the enhanced stretching vibration of O-H and N-H, and the hydrogen bonding between O-H of water molecules and C-O of amino acids, indicating the change in intramolecular or intermolecular hydrogen bonds of RBP [41]. As the absorption peaks in the amide I and amide II bands indicated the secondary structure of the protein molecule, the difference between these two peaks in the RBP emulsion gels suggested that TGase might change the secondary structure of RBP [42]. The shifts in these peaks indicated the changes in peptide and hydrogen bonding in RBP emulsion gels after TGase treatment.

### 3.5. XRD of Curcumin-Loaded RBP Emulsion Gels

The crystallinity of curcumin-loaded RBP emulsion gel was investigated by XRD and the results are shown in Figure 3B. The profile of free curcumin showed strong diffraction peaks between 5° and 30°. This was consistent with the characteristic peaks of the crystalline form of curcumin. However, the diffraction spectra of RBP–curcumin emulsion gels showed that the characteristic crystalline peaks of curcumin had disappeared, which suggested that curcumin was present in an amorphous form. This might be attributed to the entrapment of curcumin in the RBP emulsion gel, which caused crystallization inhibition and promoted the formation of amorphous complexes in the matrix. This result was consistent with the findings in curcumin-loaded rice bran albumin nanoparticles [22].

### 3.6. Appearance and SEM of RBP Emulsion Gels

**Figure 4:**
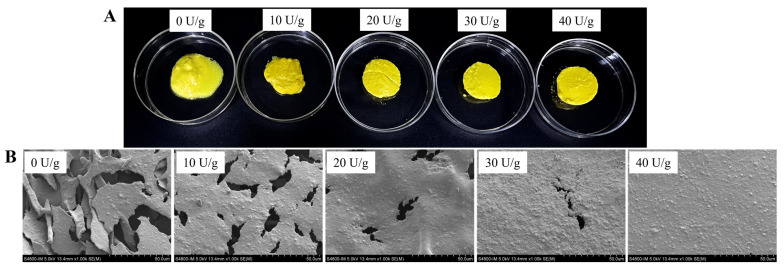
Visual images (**A**) and SEM (**B**) of RBP–curcumin emulsion gels treated with different concentrations of TGase (0–40 U/g RBP).

The visual and SEM images displayed the macrostructures and microstructures of different RBP emulsion gels, respectively. The appearance of RBP–curcumin emulsion gels is shown in Figure 4A. The texture of the sample without adding TGase was easily disrupted. The samples with the addition of TGase displayed bulk emulsion gel structures and a very intuitive protein aggregation state, along with increased integrity and homogeneity. Moreover, the emulsion gels with higher TGase concentration maintained better morphology and thereby higher plasticity. The microstructure of RBP–curcumin emulsion gels was observed by scanning electron microscope (Figure 4B). The texture of the sample without TGase cross-linking was loose and scattered, while the samples with the addition of TGase displayed tighter structures. These results confirmed the role of TGase in the formation of tight gel networks. In addition, the SEM images showed the presence of small-sized protrusions on the sample surface, which might be oil droplets.

### 3.7. Properties of Curcumin-Loaded RBP Emulsion Gels

**Figure 5:**
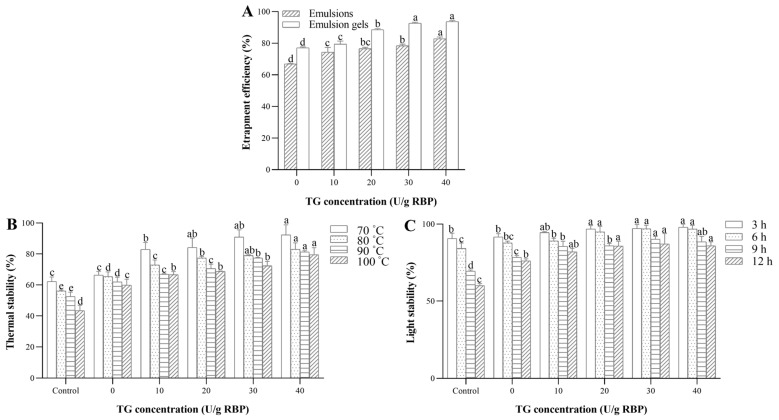
Entrapment efficiency (**A**), thermal stability (**B**), and light stability (**C**) of RBP–curcumin emulsion gels treated with different concentrations of TGase. Different letters on the bars denote significant differences among TGase concentrations for each treatment condition (*p* < 0.05).

The entrapment efficiency and environmental stability of curcumin-loaded RBP emulsion gels were measured separately (Figure 5). Curcumin entrapment efficiency increased from 77.04 ± 0.50% to 93.73 ± 0.56% after adding 40 U/g RBP TGase (Figure 5A). This was mainly due to the formation of a stable and dense gel network catalyzed by TGase. Curcumin was tightly entrapped in the gel matrix, improving the entrapment efficiency. As shown in Figure 5B,C, the stability of curcumin increased with the increasing amount of TGase. The high-strength gel effectively prevented the entrapped functional components from environmental stresses.

Thermal stability is important for the food processing industry. As shown in Figure 5B, the stability of curcumin was significantly (*p* < 0.05) affected by sample type and heating temperature. Samples treated at lower temperatures exhibited higher thermal stability than those treated at higher temperatures. This is in line with previous findings that curcumin was unstable and readily degraded to vanillin and ferulic acid at high temperatures [43]. The thermal stability of curcumin in the control group was 43.47% when heated at 100 °C for 30 min, while RBP emulsion gel increased to 59.91%. This was mainly because the RBP emulsion after ultrasonic emulsification entrapped a certain amount of curcumin, which changed it from the free state to the bound state and improved the environmental stability of the system. The addition of TGase (40 U/g) enhanced the stability of curcumin to 79.54%. Similarly, Geng et al. [29] found that transglutaminase-induced soy protein emulsion gels loaded with β-carotene exhibited higher storage stability.

As shown in Figure 5C, the content of curcumin was decreased with the increase in irradiation time. After UV irradiation for 12 h, the photo-stability of curcumin was 85.87%, 76.14%, and 60.02% in the TGase (40 U/g RBP)-induced RBP emulsion gel, RBP emulsion gel, and curcumin solution, respectively. The highest degradation of curcumin was observed in the control group because the UV radiation could transmit into the oil media directly. Both the interfacial layer and the thick structure of the emulsion gels helped to block part of the UV light, providing better protection for curcumin. In conclusion, TGase-induced RBP emulsion gels notably enhanced the stability of curcumin under light and heat conditions.

## 4. Conclusions

In this study, TGase was applied for the preparation of a novel RBP emulsion gel. TGase improved the rheological properties and textural properties of RBP emulsion gels. In addition, TGase effectively improved the entrapment efficiency and enhanced the stability of curcumin against environmental stress. TGase might affect the adsorption of the protein at the oil/water interface and promote protein aggregation during the cross-linking process. The addition of TGase improved the cross-linking structure and stability of emulsion gels, forming more uniform and compact microstructures. The curcumin-loaded RBP emulsion gels might be potentially applied in the development of functional food rich in curcumin. Thus, this study offers a technical solution for preparing higher-performance RBP emulsion gels and might provide a theoretical basis for the comprehensive utilization of rice processing by-products.

## Figures and Tables

**Table 1:** TPA parameters of RBP emulsion gels with different TGase concentrations.

Samples	Hardness/g	Chewiness/mJ	Springiness	Cohesiveness
TG-0	5.47 ± 0.59 ^d^	0.09 ± 0.01 ^e^	0.55 ± 0.04 ^d^	0.03 ± 0.01 ^c^
TG-10	9.63 ± 0.24 ^c^	0.64 ± 0.08 ^d^	0.61 ± 0.02 ^cd^	0.11 ± 0.01 ^b^
TG-20	10.57 ± 0.38 ^c^	0.96 ± 0.13 ^c^	0.62 ± 0.02 ^bc^	0.15 ± 0.02 ^a^
TG-30	11.67 ± 0.46 ^b^	1.31 ± 0.04 ^b^	0.67 ± 0.02 ^ab^	0.17 ± 0.01 ^a^
TG-40	14.73 ± 0.40 ^a^	1.76 ± 0.19 ^a^	0.73 ± 0.03 ^a^	0.16 ± 0.02 ^a^

Note: There are significant differences between different letters in the same column (*p* < 0.05).

## Data Availability

The data that support the findings of this study are available from the corresponding author upon reasonable request.
